# Perceptions of plain packaging and health warning labels for cannabis among young adults: findings from an experimental study

**DOI:** 10.1186/s12889-018-6247-2

**Published:** 2018-12-10

**Authors:** Seema Mutti-Packer, Brianne Collyer, David C. Hodgins

**Affiliations:** Department of Psychology, University of Calgary, 2500 University Drive NW, Calgary, AB T2N 1N4 Canada

**Keywords:** Cannabis, Health warnings, Young adults, Plain packaging, Canada

## Abstract

**Background:**

There is strong evidence that plain cigarette packaging and health warning labels (HWLs) reduce brand appeal and increase health knowledge. There is limited evidence examining this population-level public health approach for cannabis packaging. This issue is of particular importance in light of the recent legalization of recreational cannabis in Canada. The current study examined perceptions of plain packaging and HWLs for cannabis packages among young adults.

**Methods:**

An online experimental study was conducted with a sample of university students in Alberta, Canada (*n* = 656). Respondents were randomly assigned to view cannabis packages in one of four conditions: Condition 1: branded pack, Condition 2: plain pack (uniform color, brand imagery removed, standardized font), Condition 3: branded pack with a HWL, and Condition 4: plain pack with a HWL. Respondents in Conditions 3 and 4 viewed five text-based HWLs, each corresponding to a health effect associated with cannabis use: (1) brain development, (2) mental health issues, (3) impaired driving, (4) nonlethal overdose, and (5) addiction. After viewing packs, respondents rated packs and health warnings on various measures.

**Results:**

Branded packages without HWLs were rated as most appealing compared to all other packs (*p < 0.001* for all contrasts). No differences were found in ratings of appeal when comparing branded and plain packs with HWLs. Warning messages for cognitive development and impaired driving were rated highest on levels of perceived effectiveness, believability, and fear, whereas the addiction warning was rated among the lowest. In general, there were gaps in health knowledge related to cannabis use, however after viewing packs with warnings (compared to viewing packs without warnings) levels of health knowledge increased across all health effects (*p < 0.01* for all). Lastly, a significant majority of young adults reported they would purchase the branded pack without a HWL (39.5%), compared to all other pack types (*p < 0.05* for all contrasts). The lowest proportion of young adults reported they would purchase a plain pack with a HWL (1.1%).

**Conclusions:**

Plain packaging and health warnings may reduce brand appeal and increase health knowledge among young adults.

## Background

Worldwide, cannabis is the most widely used and cultivated illicit drug. In 2017, approximately 15% of the Canadian population (15 years and older) used cannabis during the previous year, with higher rates of use observed among youth and young adults [1]. In fact, approximately one-fifth of youth (15 to 19 years) and one-third of young adults (20 to 24 years) used cannabis in the past year [2].

In 2015, the Government of Canada committed to legalizing, regulating, and restricting access to cannabis. In 2016, a nine-member Task Force produced ‘A Framework for the Legalization and Regulation of Cannabis in Canada’ [3]. The Task Force applied a public health approach to the regulation of cannabis and proposed a number of recommendations that have the potential to minimize the harms of use. In 2017, Bill C-45 was introduced—an Act to amend the Controlled Drugs and Substances Act, the Criminal Code and other Acts. The legalization of recreational marijuana took place in October 2018.

The liberalization of cannabis use is partly due to its prevalence of use, social acceptability surrounding use, and the view that cannabis has a host of medicinal properties. While there is evidence for the use of cannabis to relieve symptoms of nausea, vomiting, loss of appetite, and pain in cancer patients [4–6], as well pain reduction in patients with fibromyalgia, arthritis, and neuropathic pain [7–9], there is also evidence that indicates that cannabis is not an entirely benign substance. Some of the adverse health effects of cannabis are dependent on the method of consumption. Combustion, one of the most common ways to consume cannabis, is associated with inflammation of large airways, increased airway resistance, and lung hyperinflation [10], and contains much of the same carcinogenic agents as tobacco smoke [11]. Regardless of the method of consumption, the adverse health effects listed below are the result of the primary psychoactive agent found in cannabis: delta-9-terahydrocannabinol, or THC.

An acute adverse health effect of cannabis use is *nonlethal overdose.* This toxic reaction occurs after an individual session of consumption. While a cannabis overdose is not known to be fatal, side effects can include severe anxiety, nausea, vomiting, psychotic episodes, or hypotension and loss of consciousness [3]. At a population level, much of the mortality and morbidity associated with cannabis use is due to traffic-related accidents involving *impaired driving* [12]. In Canada, it is estimated that 4 to 12% of vehicle-related fatalities and injuries involve cannabis [13]. The evidence also indicates that a cannabis-impaired driver is approximately twice as likely to be in a motor vehicle accident than former cannabis users, or non-users [14]. Further, evidence from Colorado indicates that after the legalization of recreational cannabis, impaired driving accidents increased, while the rates of accidents stayed the same in states that had not legalized cannabis over the same period of time [15]. In terms of *cognitive impairment,* exposure to THC during young adulthood (before the age of 25) has been shown to impair neural connectivity in adulthood [10]; an examination of regular cannabis users found decreased activity in prefrontal regions of the brain and reduced volumes of the hippocampus [16]. Cannabis use has also been associated with a variety of *mental health* issues, including increased risk of depression [17], anxiety [18, 19] and suicidal thoughts [20, 21]. Additionally, cannabis use in adolescence has been associated with the development of schizophrenia in adulthood [14]. Lastly, over the past two decades, *cannabis dependence* has been found to be the most common drug dependency after alcohol and tobacco [14]. The evidence indicates that the earlier in life and the more regular the usage, the more likely dependency will develop [10].

Despite this evidence, perceptions of the risks and harms associated with cannabis use may not be widely recognized. Previous research has shown that the adverse health effects of cannabis are not as well understood as the adverse effects of cigarettes [22, 23]. According to a 2016 survey examining perceptions of cannabis risk and harm, less than half of Canadians considered cannabis use to be more harmful than helpful to mental and physical health (42 and 41%, respectively). In addition, just over half of Canadians (58%) perceived cannabis to be addictive [24]. Thus, there is a need to address these knowledge gaps, and communicate this health information to the public.

In the domain of tobacco control, health warnings on cigarette packaging are one example of a cost-effective medium to communicate health information, given their reach and frequency of exposure at the point of purchase and during use. Health warnings have been shown to increase health knowledge, perceptions of risk, and are associated with greater motivation to quit smoking [25]. Health warnings are thought to achieve their effectiveness by eliciting a fear response, which is in line with models of health behavior including the *Health Belief Model*, and *Protection Motivation Theory* [26, 27]. In general, these models posit that emotions, such as fear, guide our decision-making; that when faced with threatening information, individuals will be motivated to engage in behaviors that protect themselves from the perceived threat. In addition to fear, the evidence from tobacco control also suggests that the believability or credibility of the message is another mechanism that underlies a warnings’ effectiveness [28, 29], and is in line with dual-process theories of attitude change such as the *Elaboration Likelihood Model* [30], which underscores the importance of engaging not only affective pathways, but also cognitive pathways [31].

In addition to communicating the health effects of cannabis, it is also critical to reduce product appeal and delay initiation, given the fact that cannabis use is concentrated among youth and young adults; a developmental period during which exposure to THC could result in detrimental developmental and neurocognitive effects. Plain packaging (removal of all brand imagery and graphics, uniform color and standardized font) is one example of a policy measure meant to reduce brand appeal among young people. In 2012, Australia became the first country in the world to implement plain packaging for cigarettes. Studies evaluating this policy have shown that brand appeal was reduced among adults and youth [32, 33], graphic health warnings became more effective among adult smokers [34], and that calls to the Quitline increased with the introduction of plain packaging [35]. Since then, France, the United Kingdom, New Zealand, Norway, Ireland, and Hungary have implemented plain packaging. Canada also recently committed to implementing plain packaging for cigarette packaging.

In line with these tobacco control strategies, the Framework report on cannabis regulation requires plain packaging, appropriate labelling (i.e., levels of THC and CBD must be listed on the package) and health warning labels on cannabis products. Specifically, the proposed regulations would require producers to include one of six health warnings on each package. Producers are also required to rotate through the six health warning messages in each calendar year.

Although plain packaging and health warnings have proven to be effective population-level interventions in tobacco control, it remains to be seen whether these policy measures will produce similar results in the context of cannabis use. Thus, the current study seeks to examine: [1] perceptions of plain (versus branded) cannabis packaging on ratings of product appeal, [2] the perceived effectiveness, believability, and levels of evoked fear of different health warning messages, [3] whether viewing health warnings increased levels of health knowledge, and [4] a behavioral measure of purchasing a cannabis package.

## Methods

Respondents were recruited through the University of Calgary Department of Psychology Research Participation System. Students accessed the Psychology Research Participation System home page, which lists all of the current available studies that they can sign up for. A large number of studies are provided as options, with brief descriptions of each study. Participants received course credit for participating in the survey, which was approximately 30 min in length. Ethical approval was obtained from the Conjoint Faculties Research Ethics Board (CFREB) at the University of Calgary. The study utilised a 5 (type of health warning) by 4 (type of packaging) mixed model design.

### Protocol

The survey was programmed with the Qualtrics online survey tool. Respondents answered a series of demographic, mental health, and cannabis use measures. Respondents were then randomly assigned to one of the four conditions: Condition 1 = branded package,^1^ Condition 2 = plain package, Condition 3 = branded package with a health warning label, 4 = plain package with a health warning label. Respondents were also asked a series of questions related to the health effects associated with cannabis use, both before and after viewing cannabis packages.

Those in Conditions 1 and 2 were shown one package with either full branding or no branding, respectively. Immediately after viewing the cannabis package in that particular condition, respondents were asked to rate the packages based on their appeal. In Conditions 3 and 4, respondents viewed five text-based health warning messages on the same cannabis package, within that particular condition (either branded or plain), for the following five health effects associated with cannabis use: [1] brain development, [2] impaired driving, [3] mental health, [4] nonlethal overdose, and [5] addiction. The wording of the five health warnings is listed in Table 1.

**Table 1:** Text-based health warnings presented on packages

Health effect	Wording on health warning
Brain development	“WARNING Regular use of this product may impair brain development in individuals under the age of 25”
Impaired driving	“WARNING Chance of motor vehicle accident almost doubles while under the influence of this product”
Mental health issues	“WARNING Regular use of this product may increase risk of mental health issues such as anxiety and depression”
Nonlethal overdose	“WARNING Overdose of this product may result in severe nausea, vomiting, and psychotic episodes”
Addiction	“WARNING Regular use of this product may be habit-forming and result in addiction or dependency”

Presentation of health warnings was counterbalanced to minimize order effects. Immediately after viewing each individual package and the corresponding health warning within that particular condition (for Conditions 3 and 4), respondents rated the packages on the following outcomes: product appeal, perceived effectiveness of the health warning, believability of the health warning, and the level of fear elicited by the health warning.

At the end of the study, respondents were shown one pack from each condition (with a generic health warning: “Use of this product may have negative impacts on your health”), and were asked “After the impending legalization of cannabis in July 2018;^2^ if you were to purchase one of the following packs, which would you choose?” This behavioral measure was meant to assess overall product appeal and purchase intention.

### Measures

#### Primary outcome measure: Product appeal

All packages were rated on their appeal, using a numeric scale, from 1 to 10, with anchors 1= “not at all” and 10 = “extremely”. “Participants were asked Overall, on a scale of 1-10, how appealing is this product?” For Conditions 3 and 4 (branded pack with a health warning label and plain pack with a health warning label, respectively), participants viewed five warning messages on the same package, each with a different health effect associated with cannabis use. For these two conditions, the *product appeal* measure was averaged across the five warnings within each experimental condition, yielding a mean score between 1 and 10.

#### Additional outcome measures

Packages in Conditions 3 and 4 (those with a health warning label) were rated on their *perceived effectiveness*, *believability*, and the level of *fear* evoked. To assess perceived effectiveness, respondents were asked “Overall, on a scale of 1-10, how effective is this health warning?” To assess believability and fear, respondents were asked “On a scale of 1 to 10, where 1 is ‘not at all’ and 10 is ‘extremely’ please indicate whether this warning message is [believable/frightening]. All three outcome measures (perceived effectiveness, believability, fear) were averaged across the five warnings within each experimental condition, yielding a mean score between 1 and 10. The measures of appeal, believability, effectiveness, and fear were adapted from previously published work [29].

#### Health knowledge

To assess health knowledge, respondents were asked the same five questions before and after viewing packages within their assigned condition: “Do you believe that cannabis use is associated with [impaired brain development]?” The same question root was asked for all five health effects (mental health issues; addiction or dependency; motor vehicle accidents; overdose causing severe nausea, vomiting, and psychotic episodes). Response options were ‘Yes’, ‘No’, ‘Unsure’, and ‘Prefer not to say’.

#### Sociodemographics, cannabis use, and mental health

Gender, age, ethnicity, cannabis use, and mental health were assessed. Current cannabis use was measured with the following: “In the last 30 days, how often did you use any cannabis products?” Response options were ‘Every day’, ‘At least once a week’, ‘At least once in the last month’, ‘Not at all’, or ‘Prefer not to say’. Those who chose ‘not at all’ were categorized as non-users; those who chose any other response option were categorized as ‘users’. Ever use was assessed by asking respondents whether they had ever used any of the following cannabis products in their lifetime: marijuana, hashish (hash), hash butane oil, cannabis oil, homemade cannabis edible, manufactured cannabis edible, shatter, wax, vaporized cannabis concentrate, cannabis tinctures. Respondents could also indicate ‘other’, if the product they had used was not listed. Cannabis use measures were adapted from the General Social Survey, and the Canadian Tobacco, Alcohol, and Drugs Survey [36, 37].

To assess mental health, the depression, anxiety, and stress scale (DASS-21) was used. The DASS-21 is a well-established 21-item self-report assessment that is made up of the depression, anxiety, and stress scales, which each contain seven items. Briefly, the depression scale assesses dysphoric mood including sadness, hopelessness, and lack of initiative. The anxiety scale assesses physical arousal including fear and panick attacks. The stress scale assesses items related to irritability, and a tendency to be easily agitated. For the complete list of scale items, please refer to [38, 39]. The DASS-21 is based on a dimensional rather than a categorical conception of psychological disorder. There are also recommended cut-off scores for different degrees of severity [38, 39].

#### Analyses

Sample size calculations were conducted to detect small mean differences in the outcome variables with 85% power, at the 5% significance level. All analyses were conducted in SPSS version 24.0. A multiple linear regression model was conducted to examine the effect of experimental condition on ratings of product appeal. To examine individual-level predictors, age, gender, ethnicity, and cannabis use were entered as covariates in the model. Interaction terms for age, gender, ethnicity, and cannabis use by experimental condition were entered into the model, individually. To determine whether there was a significant effect of the *type* of health warning message (i.e., addiction vs. brain development) on the level of each of the outcome variables: perceived effectiveness, believability, and evoked fear, repeated measures one-way analysis of variance (ANOVA) were conducted. To test the difference between levels of agreement with the five health effects associated with cannabis use, before and after the presentation of packages, McNemar χ^2^ tests were conducted. The final behavioral measure, in which respondents were asked which pack they would purchase, was meant to assess overall pack appeal. Chi-square tests were conducted to identify differences in the proportions of young adults selecting one of the four packs presented (branded pack, plain pack, branded pack with a generic health warning, plain pack with a generic health warning).

## Results

### Sample characteristics

Table 2 presents the sample characteristics. No differences in sample characteristics were observed between conditions.

**Table 2:** Sample characteristics

Characteristics	Overall, *N* = 656, % (n)	Branded *n* = 171% (*n*)	Plain *n* = 168% (*n*)	Branded HWL *n* = 163% (*n*)	Plain HWL *n* = 154% (*n*)
Gender^a^					
Male	17.5 (115)	15.2 (26)	16.1 (27)	19.6 (32)	19.5 (30)
Female	82.5 (541)	84.8 (145)	83.9 (141)	80.4 (131)	80.5 (124)
Age (mean, SD)	20.1, 3.0	20.2, 2.7	19.8, 2.8	20.1, 2.6	20.5, 3.9
Ethnicity^b^					
Caucasian	49.2 (323)	52.6 (90)	47.6 (80)	47.2 (77)	49.4 (76)
Other	50.8 (333)	47.4 (81)	52.4 (88)	52.8 (86)	50.6 (78)
Current cannabis use^c^					
Missing	1.5 (10)	2.3 (4)	1.2 (2)	1.8 (3)	0.6 (1)
Current users	15.7 (103)	14.6 (25)	15.5 (26)	13.5 (22)	19.5 (30)
Non users	82.8 (543)	83.0 (142)	83.3 (140)	84.7 (138)	79.9 (123)
Cannabis ever use^d^					
Ever use	41.3 (271)	39.8 (68)	42.3 (71)	39.9 (65)	43.5 (67)
Never use	57.3 (376)	59.1 (101)	54.8 (92)	58.9 (96)	56.5 (87)
Anxiety (mean, SD)	9.0 (8.5)	8.9 (8.7)	9.5 (8.2)	9.7 (8.6)	8.0 (8.5)
Missing	0.2 (1)	–	0.6 (1)	–	–
Normal	51.1 (335)	53.2 (91)	48.2 (81)	44.8 (73)	58.4 (90)
Mild	8.8 (58)	6.4 (11)	6.5 (11)	11.7 (19)	11.0 (17)
Moderate	18.4 (121)	18.1 (31)	20.8 (35)	21.5 (35)	13.0 (20)
Severe	7.0 (46)	6.4 (11)	7.1 (12)	9.8 (16)	4.5 (7)
Extremely severe	14.5 (95)	15.8 (27)	16.7 (28)	12.3 (20)	13.0 (20)
Stress (mean, SD)	13.0 (9.4)	13.0 (9.6)	13.4 (9.7)	13.5 (9.2)	11.8 (8.9)
Missing	0.3 (2)	0.6 (1)	0.6 (1)	–	–
Normal	63.7 (418)	64.3 (111)	59.5 (100)	61.3 (101)	70.1 (108)
Mild	10.4 (68)	9.4 (16)	8.9 (15)	12.9 (21)	10.4 (16)
Moderate	13.1 (86)	11.1 (19)	17.9 (30)	12.9 (21)	10.4 (16)
Severe	9.8 (64)	11.7 (20)	10.1 (17)	9.8 (16)	7.1 (12)
Extremely severe	2.7 (18)	2.9 (5)	3.0 (5)	3.1 (5)	1.9 (3)
Depression (mean, SD)	10.6 (10.4)	10.6 (10.8)	11.2 (10.4)	10.9 (10.2)	9.6 (10.1)
Missing	0.5 (3)	0.6 (1)	0.6 (1)	0.6 (1)	(1)
Normal	55.3 (363)	57.3 (98)	52.4 (88)	50.9 (83)	61.0 (94)
Mild	11.7 (77)	11.7 (20)	11.3 (19)	11.7 (19)	12.3 (19)
Moderate	15.1 (99)	9.9 (17)	17.9 (30)	22.7 (37)	9.7 (15)
Severe	7.6 (50)	8.2 (14)	8.9 (15)	5.5 (9)	7.8 (12)
Extremely severe	9.8 (64)	12.3 (21)	8.9 (15)	8.6 (14)	9.1 (14)

^a^Five respondents preferred not to report their gender, and were removed from the analysis due to low n

^b^Six respondents preferred not to report their ethnicity, and were removed from the analysis due to low n

^c^Five respondents preferred not to report how often they used any cannabis product in the past 30 days, and were removed from the analysis due to low n

^d^Nine respondents preferred not to report whether they ever used cannabis, and were removed from the analysis due to low n

Fourteen participants did not consent to having their data used in the study, and seven indicated that they were not paying attention during the study and did not provide appropriate data for analyses. Twenty-five respondents were removed from the dataset, due to missing data on primary measures (see footnotes in Table 2). All cases were removed on a listwise basis. In total, the data from 656 participants were used in the current study. There were 171 participants in Condition 1 (branded), 168 in Condition 2 (plain), 163 in Condition 3 (branded with a health warning), and 154 participants in Condition 4 (plain with a health warning).

As shown in Table 2, the majority of respondents (82.5%) were female. Respondents ranged from 17 to 55 years of age, with a mean age of 20 years. Most participants identified as Caucasian (49.2%), 13.6% identified as South Asian, 11.0% as Chinese, 7.6% as mixed race, 4.6% as Filipino, and 13.3% identified as each of: Aboriginal, Arab, Black, Korean, Latin American, Southeast Asian, or West Asian.

### Patterns of use

Almost half of the sample (41.3%) indicated that they had used cannabis in their lifetime and close to one-fifth of the sample (15.7%) reported current cannabis use. About a third (31.1%) of current users reported that they have used or tried cannabis for medical purposes; a majority of these respondents (53.1%) reported that the cannabis was not prescribed by a licensed physician. Current users reported significantly higher mean levels of anxiety compared to non-users (Mean = 11.4, SD = 9.9 vs. Mean = 8.6, SD = 8.2; *t* = 3.10, *p = 0.002*). No differences were found in mean stress or depression levels for current users compared to non-users (Mean = 13.9, SD = 10.3 vs. Mean = 12.8, SD = 9.2 and Mean = 11.8, SD = 10.9 vs. Mean = 10.4, SD = 10.4 respectively).

### Pack appeal ratings

A multiple linear regression model was conducted to examine overall differences in appeal ratings between experimental conditions, adjusting for age, gender, ethnicity, and cannabis use. Table 3 presents the results of the multiple linear regression model.

**Table 3:** Regression coefficients for the effect of experimental condition on product appeal ratings (*n* = 625)

	Coeff. (SE)	*p* value
Experimental condition		
Branded HWL v. Branded (ref.)	−1.40 (0.23)	*< 0.001*
Plain v. Branded (ref.)	−1.88 (0.23)	*< 0.001*
Plain HWL v. Branded (ref.)	−1.35 (0.23)	*< 0.001*
Plain HWL vs. Plain (ref.)	0.54 (0.24)	*0.022*
Plain HWL vs. Branded HWL (ref.)	0.06 (0.24)	*0.807*
Age (mean)	−0.05 (0.03)	*0.049*
Gender		
Male vs. Female (ref.)	0.67 (0.22)	*0.002*
Ethnicity		
Caucasian vs. Other (ref,)	0.001 (0.17)	*0.996*
Cannabis use		
Current use vs. Non-use (ref.)	0.78 (0.26)	*0.003*
Ever use vs. Never use (ref.)	0.78 (0.21)	*< 0.001*

The results indicated that branded packs without a health warning label were given the highest appeal ratings compared to all other pack styles (*p < 0.001* for all contrasts). Plain packs with a health warning label were rated as more appealing than plain packs without a health warning label (*p = 0.022*). No differences were observed in ratings of appeal between plain and branded packs with health warning labels. In terms of covariates, age was significant indicating that older respondents gave lower appeal ratings (*p = 0.049*). In addition, males tended to give higher appeal ratings (*p = 0.002*). Both current cannabis users and those who had ever used cannabis in their lifetime gave higher appeal ratings compared to non-users and never-users (*p = 0.003* and *p < 0.001*). The interaction terms of experimental condition by age, gender, ethnicity, and cannabis use, were not significant and were removed from the final model.

### Perceived effectiveness, believability, and evoked fear

Repeated measures one-way ANOVAs were conducted to determine whether there was a significant effect of the type of health warning message on the level of each of the outcome variables: perceived effectiveness, believability, and evoked fear. A significant effect was found for all three outcome variables (F_*(df = 4)*_ = 40.54, *p < 0.001*; F_*(df = 4)*_ = 26.63, *p < 0.001*; F_*(df = 4)*_ = 48.19, *p < 0.001*, respectively).

Table 4 presents the mean overall ratings of perceived effectiveness, believability, and evoked fear for each health warning message, as well as significant differences between each warning message theme. For ratings of perceived effectiveness, the brain development health warning was rated highest, whereas the addiction warning was rated lowest; all other warnings were rated similarly. For ratings of believability and fear, both the brain development and impaired driving warnings were rated highest.

**Table 4:** Overall mean ratings of perceived effectiveness, believability, and evoked fear for health warning messages

	Health warning messages				
	Brain development	Impaired driving	Overdose	Mental health	Addiction
Perceived effectiveness	5.3 (2.2)^a^	4.7 (2.2)^b^	4.5 (2.2)^b^	4.5 (2.1)^b^	3.9 (2.0)^c^
Believability	6.3 (2.2)^a^	6.2 (2.2)^a^	5.6 (2.3)^b^	5.4 (2.3)^b^	5.3 (2.4)^b^
Fear	5.6 (2.6)^a^	5.4 (2.5)^a^	4.9 (2.5)^b^	4.5 (2.4)^c^	4.0 (2.3)^d^

Numbers in the table are mean ratings; higher numbers indicate higher mean ratings. Different letters denote significant differences of ratings, based on paired t-tests, where *p* < 0.01

No differences were observed in ratings of health warnings between the plain and branded package conditions, thus, the ratings were combined across conditions

### Health knowledge

McNemar χ^2^ tests were conducted to test the difference between levels of agreement with the five health effects associated with cannabis use, before and after the presentation of cannabis packages with no health warnings (Conditions 1, 2) and cannabis packages with health warnings (Conditions 3, 4). As shown in Table 5, viewing cannabis packages with health warnings significantly increased knowledge across all health effects.

**Table 5:** Percent change in agreement with five health effects associated with cannabis use

	No HWL (*n* = 336)			With HWL (*n* = 313)		
	Pre %	Post %	% change	Pre %	Post %	% change
Brain development	61.5	60.7	−0.8	61.3	70.6	+ 9.3***
Impaired driving	66.6	67.2	0.6	70.3	76.5	+ 6.2**
Overdose	48.1	48.1	–	41.8	57.3	+ 15.5***
Mental health	48.4	48.1	−0.3	44.6	56.4	+ 11.8***
Addiction	54.1	57.1	+ 3.0*	54.0	59.6	+ 5.6**
	Branded with HWL (*n* = 163)			Plain with HWL (*n* = 152)		
	Pre	Post	% change	Pre	Post	% change
Brain development	63.2	72.4	+ 9.2**	58.4	66.9	+ 8.5**
Impaired driving	66.3	74.8	+ 8.5**	74.0	77.3	+ 3.3
Overdose	39.3	56.4	+ 17.1***	44.2	57.1	+ 12.9***
Mental health	41.7	55.2	+ 13.5***	47.4	56.5	+ 9.1**
Addiction	54.6	57.6	+ 3.0	52.6	59.7	+ 7.1

% change column represents the difference in the percentages of respondents agreeing with the health effect associated with cannabis use, before and after viewing packages without health warnings (Conditions 1 and 2) and with health warnings (Conditions 3 and 4), and after viewing branded packages with health warnings (Condition 3) and plain packages with health warnings (Condition 4). Positive numbers indicate an increase in agreement with the health effect. *p < 0.05, **p < 0.01 ***p < 0.001

Next, to assess whether there was an additive effect of including the health warning on plain packaging (vs. branded), the percent change in health knowledge was compared between branded packages with health warnings (Condition 3), and plain packages with health warnings (Condition 4). As shown in Table 5, an additive effect of adding the health warning on plain packaging was not found.

### Behavioral measure of purchasing a pack

As a final task, respondents were shown one pack from each condition (for packs with a health warning, a generic warning was used), and were asked “After the impending legalization of cannabis in July 2018^2^; if you were to purchase one of the following packs, which would you choose?” About one-third of respondents indicated that they would not purchase cannabis at all (33.1%). A significant proportion of respondents indicated that they would choose the branded pack without a health warning label (39.5%), compared to plain packs without a health warning label (10.8%), branded packs with a health warning label (9.8%), and finally plain packs with a health warning label (1.1%) (*p < 0.05* for all contrasts).

## Discussion

The findings regarding the efficacy of health warnings and plain packaging mirror those found in tobacco control. Overall, viewing packages with plain packaging and health warnings increased levels of health knowledge across all health effects, and reduced product appeal.

Interestingly, the plain package *with* a health warning was given *higher* appeal ratings compared to the plain package *without* a health warning. This was the opposite of what was expected and could partly be due to the fact that the adverse effects of cannabis use may not be widely understood [22–24]. For example, in the current sample, less than half (45.2%) of respondents agreed that cannabis use was associated with mental health issues such as anxiety and depression, which may have lessened the credibility of the warnings. If this were the case, the plain packaging (uniform color, no brand imagery) may have increased the salience and novelty of the health warnings, and increased attention and interest in the packages, leading to greater appeal ratings. In the remaining analyses, health warnings on plain packages were also not rated as more effective, believable nor fear-inducing than those on branded packs. Together, these findings indicate there was no additive effect of adding a health warning to plain packaging for the outcome measures of product appeal, perceived effectiveness, believability, nor fear, in the current study. A similar pattern was also found when considering levels of health knowledge; there was no additive effect when combing the health warning label with plain packaging.

However, when considering the behavioral measure of purchasing a cannabis pack, the combination of plain packaging and a health warning label did *decrease* the likelihood that respondents would purchase that pack. A similar study protocol was carried out in Brazil and the UK for tobacco. In these studies, instead of being asked which pack they would purchase, respondents were told they were being given a pack. In Brazil, it was found that respondents were three times more likely to select branded cigarette packs compared to plain [40]. In the UK, about 95% of respondents selected a branded pack, whereas only 5% selected the plain pack [41].

The findings from the current study also highlight gaps in health knowledge across most health effects associated with cannabis use, particularly for: *nonlethal overdose*, *mental health*, and *addiction* warning messages. However, it was also found that viewing packs with health warnings increased health knowledge across all health effects, when compared to packs without health warnings, and is particularly important when considering the *motivational hypothesis* [42, 43]. The motivational hypothesis posits that risk perceptions predict behavioral intentions—in this view, an increase in risk perceptions may lead to an increase in preventive behavior or a decrease in risky behavior. Recent longitudinal research examining directional influences of risk perceptions on tobacco, alcohol, and cannabis use found evidence for this hypothesis; in general, changes in risk perception predicted changes in future use [44]. Similarly, cross-sectional studies from tobacco control have demonstrated that smokers with greater knowledge of the health risks of smoking were more likely to intend to quit and were more successful in their quit attempts [45, 46]. Together, these findings suggest that health warnings for cannabis products may help communicate risk and increase health knowledge.

In terms of health warning label content, the warnings that were given the highest ratings of effectiveness, believability, and which elicited the greatest levels of fear— *impaired driving* and *brain development*—were also endorsed by the greatest proportion of respondents as health effects associated with cannabis use, prior to viewing health warnings (68.4 and 61.4%, respectively). Similarly, the health warning for addiction received among the lowest ratings of effectiveness, believability, and evoked fear, in addition to a smaller proportion of respondents endorsing it as a health effect associated with cannabis use, prior to viewing health warnings (54.0%). These findings suggest that health effects that are more widely endorsed may increase perceptions of effectiveness, believability and levels of fear evoked when viewing health warnings, highlighting the need to communicate this health information to the public.

### Limitations

As in any survey research, there is the possibility that respondents may not be honest in their responses, particularly when asked about the use of illicit substances, as in the current study. To mitigate this possibility, respondents were assured at the start of the online survey that all responses would be confidential and anonymous—that no personal identifiers would be assigned to the data collected. With that said, it is still possible that respondents may choose to answer in a socially desirable way, resulting in an underestimation of current cannabis use. Furthermore, this university-based sample of young adults is not nationally representative, however with respect to cannabis use, young adults are of primary interest considering higher rates of use in this population [1]. Although this sample was not nationally representative, and the potentially sensitive topic area may have resulted in an underestimation of cannabis use, the patterns of use in the current study are very similar to those based on national samples. In the current sample, 15.7% indicated they had used cannabis sometime in the past 30 days, compared to 17.9% of students from the 2016 National College Health Assessment Survey, a convenience sample of 41 post-secondary institutions in Canada. In addition, 57.3% of respondents in the current study indicated they never used cannabis, compared to 58.4% of students in the previously mentioned survey [47].

The study was designed after the Canadian Framework report was released, but prior to the release of the proposed regulations, which provide a detailed description of packaging and labelling requirements for cannabis products. Thus, the packaging and labelling used in the current study, while similar in some respects to the current regulations, are not identical. For example, the labels in the current study do not include a standardized cannabis symbol, nor do the plain packages include one other brand element (logo/slogan/graphic) in addition to the brand name, as outlined in the regulations. However, the warning message content in the current study is based on an extensive review of the literature, and also mirrors the content areas in the proposed regulations, with the exception of the pregnancy-related content, and content related to the harmfulness of cannabis smoke.

Finally, the warnings tested in the current study were text-based, as required by the Government of Canada. A wealth of evidence from tobacco control has shown the superiority of pictorial, graphic warnings compared to text-only [25, 48]. Future research should seek to examine the efficacy of including graphic images alongside text-based warnings.

### Conclusions

Overall, the findings suggest that plain packaging and health warnings have the potential to reduce product appeal and increase health knowledge among young adults.

## Abbreviations

ANOVA: Analysis of variance
CBD: Cannabidiol
DASS: The Depression Anxiety Stress Scales
HWL: Health warning label
THC: Tetrahydrocannabinol

## Footnotes

1 One locally available brand was used in the study.

2 At the time the study was conducted, the proposed date of legalization was set for July 2018. As of publication, the date for legalization was postponed to October 17, 2018.
